# Institutional delivery service utilization and associated factors in Ethiopia: a systematic review and META-analysis

**DOI:** 10.1186/s12884-020-03032-5

**Published:** 2020-06-15

**Authors:** Adane Nigusie, Telake Azale, Mezgebu Yitayal

**Affiliations:** 1 Department of Health Education and Behavioral Sciences, Institute of Public Health, College of Medicine and Health Sciences, University of Gondar, Gondar, Ethiopia; 2 Departement of Health Systems and Policy, Institute of Public Health, College of Medicine and Health Sciences, University of Gondar, Gondar, Ethiopia

**Keywords:** Skilled birth attendance, Women, Facility-based delivery

## Abstract

**Background:**

There is wide variation in the utilization of institutional delivery service in Ethiopia. Various socioeconomic and cultural factors affect the decision where to give birth. Although there has been a growing interest in the assessment of institutional delivery service utilization and its predictors, nationally representative evidence is scarce. This study was aimed to estimate the pooled national prevalence of institutional delivery service utilization and associated factors in Ethiopia.

**Methods:**

Studies were accessed through PubMed, Cochrane library, Web of Science, and Google Scholar. The funnel plot and Egger’s regression test were used to see publication bias, and I-squared statistic was applied to check heterogeneity of studies. A weighted Dersimonian laired random effect model was applied to estimate the pooled national prevalence and the effect size of institutional delivery service utilization and associated factors.

**Result:**

Twenty four studies were included in this review. The pooled prevalence of institutional delivery service utilization was 31% (95% Confidence interval (CI): 30, 31.2%; I^2^ = 0.00%). Attitude towards institutional delivery (Adjusted Odd Ratio (AOR) = 2.83; 95% CI 1.35,5.92) in 3 studies, maternal age at first pregnancy (AOR = 3.59; 95% CI 2.27,5.69) in 4 studies, residence setting (AOR = 3.84; 95% CI 1.31, 11.25) in 7 studies, educational status (AOR = 2.91;95% 1.88,4.52) in 5 studies, availability of information source (AOR = 1.80;95% CI 1.16,2.78) in 6 studies, ANC follow-up (AOR = 2.57 95% CI 1.46,4.54) in 13 studies, frequency of ANC follow up (AOR = 4.04;95% CI 1.21,13.46) in 4 studies, knowledge on danger signs during pregnancy and benefits of institutional delivery (AOR = 3.04;95% CI 1.76,5.24) in 11 studies and place of birth of the elder child (AOR = 8.44;95% CI 5.75,12.39) in 4 studies were the significant predictors of institutional delivery service utilization.

**Conclusion:**

This review found that there are several modifiable factors such as empowering women through education; promoting antenatal care to prevent home delivery; increasing awareness of women through mass media and making services more accessible would likely increase utilization of institutional delivery.

## Background

Globally, a total of 13.6 million women have died due to complications during and following pregnancy and childbirth between 1990 and 2015 [1]. Majority of maternal health complications and deaths occurred in low and middle income countries. Three-fourth of the deaths were due to preventable direct obstetric complications [2–6]. Institutional delivery service utilization ensures safe birth, reduces both actual and potential complications and maternal death, and increases the survival `of mothers and newborns. Most deliveries in developing countries occur at home without skilled birth attendants [1, 7] even though many of the developing countries tried their best to optimize key and effective maternal health interventions to improve maternal health [8].

Behavioral intention (BI) is an indication of a person’s readiness to perform a given behavior or action. Intention of pregnant women to utilize institutional delivery service is affected by socio-demographic variables, household monthly income, health institution, mother’s occupation and husband’s occupation. Psychosocial variables such as perceived susceptibility to pregnancy and birth complications, perceived barriers to utilize institutional delivery service, self-efficacy, being able to make decision, and, being informed where to deliver are predictors of the probability of giving birth at health institution [9]. In 2014, about 71% of women delivered with the support of a skilled birth attendant which is better as compared to 59% in 1990 worldwide.

On the other hand, there is little progress in closing the gap in antenatal care between urban and rural women [10]. The risk of maternal death is now increasingly concentrated in sub-Saharan Africa as a result of high fertility rates combined with inadequate access to quality antenatal care and skilled attendance at birth. Ending preventable maternal, new-born and child deaths in the Sustainable Development Goals (SDG) will be essential to bring about significant improvements in levels of coverage, and quality of care provided before, during and after birth. A woman who receives delivery service with professional assistance has critical health benefits for both the mother and child [11]. Since most of the maternal deaths and complications occur around the time of delivery, institutional delivery with the help of skilled birth attendance is one of the most important interventions in reducing maternal mortality and complications [7]. An estimated 13–33% of maternal deaths could be reduced by skilled attendance during labor, delivery and the early post-partum period [12]. The skilled attendance at birth (SBA) rate is extremely low in many settings, especially in sub-Saharan African and South Asian countries. A finding from Uttarakh, India shows that only 33% of the study participants delivered their index child at health facility [13].On the other hand a finding from Ethiopia shows that only 11.3% women delivered their index child at health facility [14].

In sub-Saharan Africa, a woman’s risk of dying from treatable or preventable complications of pregnancy and childbirth over the course of her lifetime is 1 in 22, compared to 1 in 7300 in the developed regions [15]. Even though the 2016 Ethiopian Demographic and Health Survey (EDHS) shows reduction in maternal mortality in Ethiopia compared to the 2011 EDHS, it is still one of the highest figures, accounting for 412 deaths per 100,000 live births [16].

**Fig. 1:**
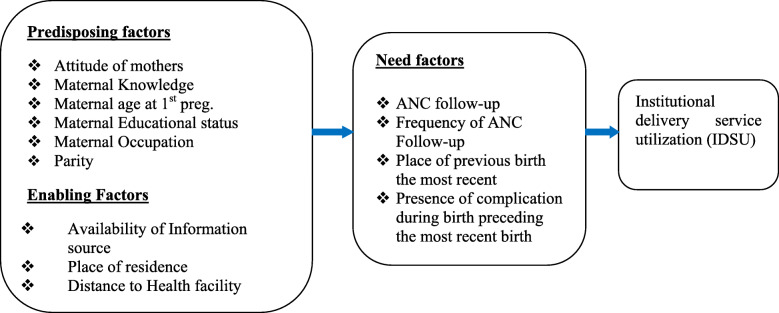
Conceptual framework of factors associated with the utilization of institutional delivery services in Ethiopia

The health care seeking behavior of a woman regarding institutional delivery affects her chances of accessing and receiving institutional delivery care, particularly in developing countries where an equitable health care system is yet to be set up. Mothers with low health care seeking behavior regarding institutional delivery have a disproportionate burden of maternal deaths. Therefore, this systematic review and meta-analysis aimed, to estimate the pooled prevalence, and the effect size of associated factors of institutional delivery service utilization, in the Ethiopian context, thereby making the available evidence accessible to decision makers **(**Fig. 1**).**

## Methods

The review protocol was developed based on the 2015 Preferred Reporting Items for Systematic Reviews and Meta-Analyses (PRISMA-P) [17], and submitted and published in PROSPERO with an ID of CRD42019124210. The database was searched for the same systematic review to avoid duplications. The website (http://www.library.UCSF.edu), and PROSPERO and Cochrane/Wiley library were explored to confirm whether previous systematic review or meta-analysis exists.

### Inclusion and exclusion criteria

Studies with cross-sectional, case-control, and cohort designs were included. Those studies that had reported the prevalence and/or a minimum of one associated factor with institutional delivery service utilization and published in English were considered [18]. Studies conducted altogether regions of Ethiopia (Amhara, Oromiya, SNNPR, Tigray, Bie.Guz, Afar, Gambiela, Somalia, Harari, Dirie Dawa and Addis Abeba) from 2010 to December 30/2018 were considered for the review. The main reason for selecting 2010 as a year of the starting point was that home delivery free (HDF) strategy (including free maternity services, availability of ambulance, etc.) was not available altogether parts of Ethiopia before this. Citations without abstract and/ or full-text, anonymous reports, editorials, and qualitative studies were excluded from the analysis. We used PICO mnemonic (population, intervention, comparator and outcome) to construct a clear and meaningful review objective/question: **Population/Participants**: reproductive aged women (15–49 years) who gave birth 2 years prior to the conduct of the study and who live in Ethiopia; **Interventions/Exposure:** Institutional delivery; and **Outcome:** Institutional delivery service utilization.

### Search strategy and information sources

All studies were systematically searched through electronic databases including PubMED, Cochrane library, Web of Science, Google, and Google Scholar.The authors were contacted for the articles with incomplete reported data [18]. The core search terms and phrases we used were "Institutional delivery" "health facility delivery" "service" "factors associated" "reproductive age women" "15–49 year’s women" and "Ethiopia". Different Boolean operators were used in order to develop the search strategies.

Particularly, to fit the advanced PubMed database, the following search strategy was applied:

[(Institutional delivery) [All Fields] OR Institutional delivery [MeSH Terms])] AND [service) [All Fields] OR service [MeSH Terms])] AND [factors) [All Fields] OR factors [MeSH Terms])] AND [reproductive age women) [All Fields] OR reproductive age women [MeSH Terms]) OR 15–49 years women) [All Fields] OR 15–49 years women [MeSH Terms]] AND [Ethiopia].

### Data extraction

The data were extracted by two independent reviewers’ using a structured data extraction form. When variations of extracted data between the reviewers were observed, the phase was repeated. If discrepancies between data extractors continued, a third reviewer was involved. The name of the first author and year, the study region, the study design, the target population, the sample size, prevalence of institutional delivery service utilization, and Adjusted Odd Ratio (AOR) with 95% Confidence interval (CI) of the associated factors with institutional delivery were collected.

### Study selection

**Fig. 2:**
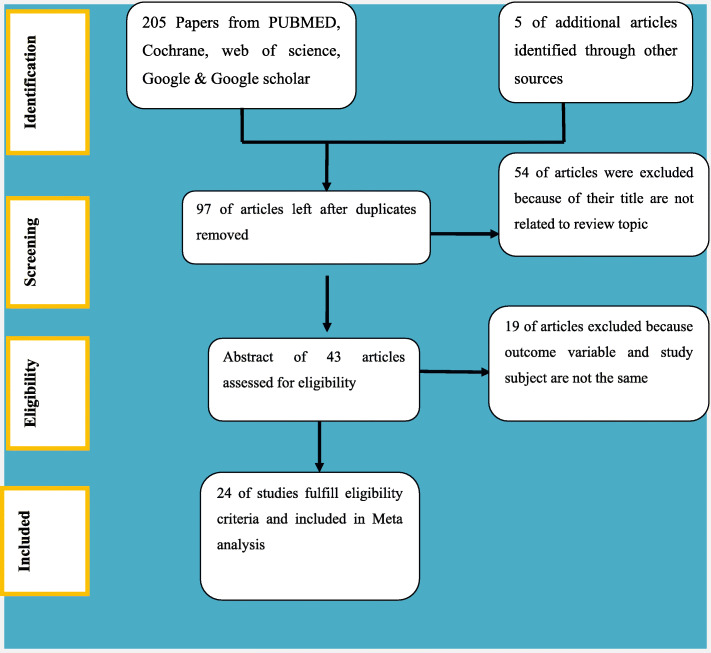
Study selection process

All the retrieved studies were exported to reference manager software, Endnote version 7 to get rid of duplicate studies [18]; during which 203 articles were removed. Three independent reviewers screened the title and abstract. The disagreement between the reviewers was handled based on established article selection criteria. Fifty four (54) articles/study titles and abstracts did not fit or were not related with our review were removed from full text assessment. Forty three (43) articles were going for full text assessment of the eligibility and 19 of them were excluded from synthesis because the outcome variable and study participants were not the same as our review objectives. Twenty four (24) studies were included within the prevalence and/or associated factor estimation **(**Fig. 2**).** Three independent authors (AN, TA, MY) conducted the abstract and full-text review.

### Quality assessment/critical appraisal

The quality of individual studies was appraised by three independent authors using the Joanna Briggs Institute (JBI) quality appraisal checklist [19, 20]. The disagreement was resolved by the interference of a third reviewer [18].

**Cohort studies** were appraised using the following items: Similarity of groups; Similarity of exposure measurement; Validity and reliability of measurement; Identification of confounder; Strategies to deal with confounder; Appropriateness of groups/participants at the start of the study; Validity and reliability of outcome measured; Sufficiency of follow-up time; Completeness of follow-up or descriptions of reason for loss to follow-up; Strategies to address incomplete follow-up; and Appropriateness of statistical analysis. Studies got 50% and above of the quality scale were considered low risk. We had only one cohort/follow up study with a quality score of 81.2% which was included in the study.

**Case-control studies** were appraised using the following items: Comparable groups; Appropriateness of cases and controls; Criteria to identify cases and controls; Standard measurement of exposure; Similarity in measurement of exposure for cases and controls; Handling of confounder; Strategies to handle confounder; Standard assessment of outcome; Appropriateness of duration for exposure; and Appropriateness of statistical analysis. Studies got 50% and above of the quality scale were considered low risk. We had only one case control study with a quality score of 65%which was included in the study.

**Cross-sectional studies** were appraised using the following items: Inclusion criteria; Description of study subject and setting; Valid and reliable measurement of exposure; Objective and standard criteria used; Identification of confounder; Strategies to handle confounder; Outcome measurement; and Appropriate statistical analysis. Studies were considered low risk when it scored 50% and above of the quality assessment indicators. We had 22 cross-sectional studies and all got a score of above 50% of the quality scale, which is low risk and can be included in the study.

### Outcome measurement

Institutional delivery service utilization was considered, when women reported that they gave their most recent birth (within the last 2 years) at a health institution.

### Data synthesis and analysis

The data synthesis was done in a clear and detailed descriptive summary of the included studies via tabulating the name of author, year of study or year of publication, study design, and number of study participants.

The data entry and statistical analysis were carried out using STATA-11 software. Tables and figures were used to summarize the selected studies and results descriptively. We also implemented a meta-analysis of studies that provided a comparable classification of the determinants or exposures and the outcome variables.

**Fig. 3:**
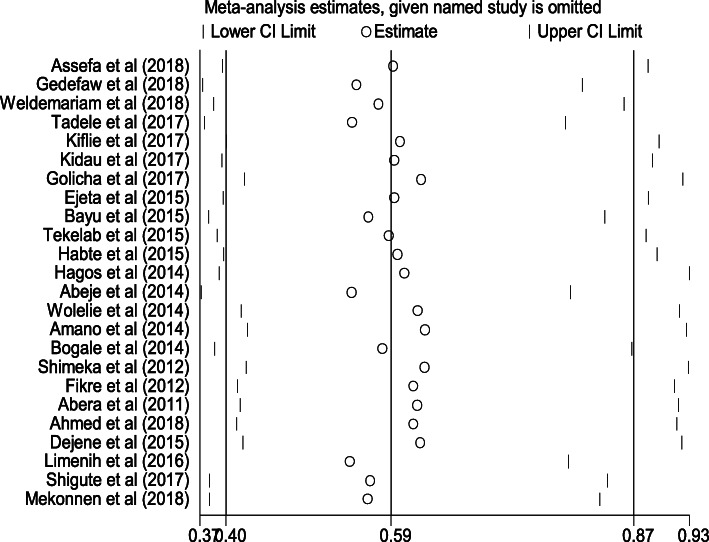
The sensitivity analysis showed the pooled proportion when the studies omitted step by step

For the meta-analysis, we considered estimates of adjusted odds ratio with the confidence interval (CI) as the measure of association. The overall effect (pooled estimates of the magnitude and the factors) of institutional delivery service utilization was estimated using a random effect model and measured by the prevalence rates and odds ratio with 95% CI. We selected the random effect model because of heterogeneity due to difference in the study design and study regions. To determine heterogeneity among studies, we calculated the I^2^ statistic, which describes the percentage of total variation among studies due to heterogeneity rather than chance. Statistical heterogeneity assessed using Forest plot, Cochrane’s Q statistic (*P* value < 0.1) and I square tests (> 50%). Heterogeneity of studies was quantified using the I-squared statistic, in which 25, 50, and 75% represented low, moderate and high heterogeneity respectively [21]. Pooled analysis was conducted using a weighted DerSimonian and Laird random-effects model [22]. Publication bias was checked by funnel plot and more objectively through Egger’s regression test [23]. Subgroup analysis was done by the study setting (region), design, and year of publication. Sensitivity analysis was employed to see the effect of single study on the overall estimation **(**Fig. 3**).** Variations through time were checked by conducting time trend analysis [18].

## Results

### Study characteristics and study participants

Based on the search strategy we have been designed at the protocol development stage we have retrieved 104 studies from PubMed, 23 studies from Cochrane library, 16 studies from Web of Science, 55 studies from Google, 69 studies from Google Scholar and 05 studies from other sources. After removing duplicated studies, 97 studies remained. Finally, 43 studies were screened for full-text review and 24 studies were included in the prevalence and/ or associated factor analysis **(**Fig. 2**).** Seven studies were found in Amhara region [24–30], Nine in Oromia [31–39], two in Afar [40, 41], one in Bienishangul-Gumuz [42],three in Southern Nation Nationalities and Peoples region (SNNPR) [43–45],one in Gambiela [46], and one in Tigray and Oromiya [47].

The design of 22 of the studies examined was cross-sectional, one prospective follow up, and one case control. The year of publication ranged from 2010 to 2018, five studies were published in 2018, 11 studies were published between 2015 and 2017, and eight studies were between 2011 and 2014. Table 1 summarizes the characteristics of the 19,969 women who gave birth within the 2 years preceding the survey and among which 6672 women gave birth in health institution. All twenty four studies had an enrollment period exclusively after 2010, when institutional delivery service utilization with at least one factor becomes available. All papers stated the proportions of institutional delivery service utilization. A large proportion of the participants (69%) in these studies did not give birth at health facility for their last birth (Table 1).

**Table 1:** Characteristics and quality status of the studies

S.n	Reference	Location	objective	Study design	Participants/Subjects	Sample size	Key finding; Prevalence of institutional delivery (%)	Quality status by using Joanna Briggs Institute checklist
1	Luelseged Assefa, Mussie Alemayehu and Ayal Debie, Magnitude of institutional delivery service utilization and associated factors among women in pastoral community of Awash Fentale district Afar Regional State, Ethiopia.*BMC Res Notes (2018) 11:162*	Afar	*To assess institutional delivery service utilization and associated factors among women in pastoral community of Awash Fentale district, Ethiopia.*	CS	women who gave birth in the last one years	423	35.2	Low risk
2	Getnet Gedefaw, Eskeziaw Abebe, Rebka Nigatu, Bethelihem Mesfin, and Amanuel Addisu,Institutional Delivery Service Utilization and its Factors Influencing Among Mothers Who Gave Birth in Woldia Town, Ethiopia. A Community-Based Cross-Sectional Study:.Gynecol Obstet (Sunnyvale) 2018, 8:9	Amhara	*To assess the prevalence and factors associated with institutional delivery service utilization of childbearing age group women who gave birth in Woldia town.*	CS	women who gave birth in the last one years	360	74.7	Low risk
3	Solomon Weldemariam, Amare Kiros and Mengistu Welday, Utilization of institutional delivery service and associated factors among mothers in North West Ethiopian.*BMC Res Notes (2018) 11:194*	Bie.Guz	To assess institutional delivery and its associated factors in Benishangul-Gumez region, North-West of Ethiopia	CS	women who gave birth in the last one years	427	51.1	Low risk
4	Niguse Tadele and Tafesse Lamaro, Utilization of institutional delivery service and associated factors in Bench Maji zone, Southwest Ethiopia: community based, cross sectional study. BMC Health Services Research (2017) 17:101	SouthernNation Nationality and Peoples Region	*To assess utilization of institutional delivery and related factors in Bench Maji zone, Southwest Ethiopia.*	CS	women who gave birth in the last two years	765	78.3	Low risk
5	Dereje Kifle, Telake Azale, Yalemzewod Assefa Gelaw and Yayehirad Alemu Melsew, Maternal health care service seeking behaviors and associated factors among women in rural Haramaya District, Eastern Ethiopia: a triangulated community-based cross-sectional study. Reproductive Health (2017) 14:6	Oromiya	*To assess the maternal health care seeking behavior and associated factors of reproductive age women in rural villages of Haramaya district, East Ethiopia.*	CS	women who gave birth in the last two years	561	28.7	Low risk
6	Sewnet Kidanu, Genet Degu and Tenaw Yimer Tiruye, Factors nfluencing institutional delivery service utilization in Dembecha district, Northwest Ethiopia: A community based cross sectional study. Reproductive Health (2017) 14:98	Amhara	*T*o assess factors influencing institutional delivery service utilization in Dembecha district, Northwest Ethiopia.	CS	women who gave birth in the last two years	674	34.0	Low risk
7	Wako Golicha Wako and Dejene Hailu Kassa, Institutional delivery service utilization and associated factors among women of reproductive age in the mobile pastoral community of the Liban District in Guji Zone, Oromia, Southern Ethiopia: a cross sectional study. BMC Pregnancy and Childbirth (2017) 17:144	Oromiya	*T*o assess utilization of institutional delivery and associated factors among women of reproductive age in the mobile pastoral community of the Liban District in Guji zone, Oromia, Ethiopia.	CS	women who gave birth in the last two years	791	13.9	Low risk
8	Eshetu Ejeta, Tadele Nigusse, Determinants of Skilled Institutional Delivery ServiceUtilization among Women Who Gave Birth in the Last 12 Months in Bako District, Oromia, Ethiopia, 2012/13(Case-Control Study Design). Journal of Gynecology and Obstetrics 2015; 3(2): 36–42	Oromiya	*To* assess factors affecting utilization of skilled institutional delivery services among women who gave birth in the last 12 months preceding the study in Bako woreda, West Shoa Zone, Ethiopia, 2012/13.	CC	women who gave birth in the last one years	380	34.2	Low risk
9	Hinsermu Bayu, Mulatu Adefris2, Abdella Amano and Mulunesh Abuhay, Pregnant women’s preference and factorsassociated with institutional delivery service utilization in Debra Markos Town, North West Ethiopia: a community based follow up study. BMC Pregnancy and Childbirth (2015) 15:15	Amhara	To assess Pregnant women’s preference and factors associated with institutional delivery service utilization in Debra Markos Town, North West Ethiopia.	Cohort	2nd and 3rd trimester pregnant women	393	62.3	Low risk
10	Tesfalidet Tekelab, Birhanu Yadecha and Alemu Sufa Melka, Antenatal care and women’s decision making power as determinants of institutional delivery in rural area of Western Ethiopia. *BMC Res Notes (2015) 8:769*	Oromiya	to determine the prevalence of institutional delivery service utilization and associated factors in rural area of East Wollega Zone, Western Ethiopia.	CS	women who gave birth in the last two years	798	39.7	Low risk
11	Feleke Habte and Meaza Demissie, Magnitude and factors associated with institutional delivery service utilization among childbearing mothers in Cheha district, Gurage zone, SNNPR, Ethiopia: a community based cross sectional study. BMC Pregnancy and Childbirth (2015) 15:299	Southern Nation Nationality and Peoples Region	*To* measure the prevalence and to identify factors associated with institutional delivery service utilization among childbearing mothers in Cheha District, SNNPR, Ethiopia.	CS	women who gave birth in the last two years	816	31.0	Low risk
12	Seifu Hagos, Debebe Shaweno, Meselech Assegid, Alemayehu Mekonnen, Mesganaw Fantahun Afework and Saifuddin Ahmed, Utilization of institutional delivery service at Wukro and Butajera districts in the Northern and South Central Ethiopia. BMC Pregnancy and Childbirth 2014, 14:178	Tigray&Oromiya	to determine the magnitude and identify factors affecting delivery at health institution in two districts in Ethiopia.	CS	women who gave birth in the last two years	4949	25.0	Low risk
13	Gedefaw Abeje, Muluken Azage and Tesfaye Setegn, Factors associated with Institutional delivery service utilization among mothers in Bahir DarCity administration, Amhara region: a community based cross sectional study. Reproductive Health 2014, 11:22	Amhara	*T*o assess factors associated with institutional delivery service use among mothers in Bahir Dar city administration	CS	women who gave birth in the last one years	481	78.8	Low risk
14	Alemaw Wolelie, Mekonnen Aychiluhm,Worku Awoke, Institutional delivery service utilization and associated factors in Banja District, Awie Zone, Amhara Regional Sate, Ethiopia. Open Journal of Epidemiology, 2014, 4, 30–35	Amhara	*To* assess institutional delivery service utiliza- tion and associated factors in Banja District, Amhara region, Ethiopia, 2013	CS	women who gave birth in the last two years	394	15.7	Low risk
15	Abdella Amano, Abebaw Gebeyehu and Zelalem Birhanu, Institutional delivery service utilization in Munisa Woreda, South East Ethiopia: a community based cross-sectional study. BMC Pregnancy and Childbirth 2012, 12:105	Oromiya	*T*o determine the level of institutional delivery service utilization and associated factors.	CS	women who gave birth in the last one years	855	12.3	Low risk
16	Daniel Bogale Odo1, Desalegn Markos Shifti, Institutional delivery service utilization and associated factors among child bearing age women in Goba Woreda, Ethiopia. Journal of Gynecology and Obstetrics 2014; 2(4): 63–70	Oromiya	Assessing level of institutional delivery service utilization and associated factors among mothers who gave birth during the last twelve months prior to this study.	CS	women who gave birth in the last one years	562	47.0	Low risk
17	Alemayehu Shimeka Teferra, Fekadu Mazengia Alemu and Solomon Meseret Woldeyohannes Institutional delivery service utilization and associated factors among mothers who gave birth in the last 12 months in Sekela District, North West of Ethiopia: A community - based cross sectional study. BMC Pregnancy and Childbirth 2012, 12:74	Amhara	*To* assess factors affecting institutional delivery service utilization among mothers who gave birth in the last 12 months in Sekela District, Amhara Region, Ethiopia.	CS	women who gave birth in the last one years	371	12.1	Low risk
18	Addis Alem Fikre and Meaza Demissie,Prevalence of institutional delivery and associated factors in Dodota Woreda (district), Oromia regional state, Ethiopia. Reproductive Health 2012, 9:33	Oromiya	*To* determine the prevalence of institutional delivery and understand the factors associated with institutional delivery in Dodota, Woreda, Oromia Region.	CS	women who gave birth in the last two years	506	18.2	Low risk
19	Mulumebet Abera, Abebe G/mariam, Tefera Belachew,PREDICTORS OF SAFE DELIVERY SERVICE UTILIZATION IN ARSI ZONE, SOUTH-EAST ETHIOPIA. Ethiop J Health Sci. Vol. 102 21, Special Issue August, 2011	Oromiya	*To assess the predictors of safe delivery service utilization in Arsi Zone, Southeast Ethiopia.*	CS	women who gave birth in the last one years	1074	16.4	Low risk
20	Mohammed Ahmed, Meaza Demissie, Araya Abrha Medhanyie, Alemayehu Worku, Yemane Berhane,Utilization of Institutional Delivery Service in a Predominantly Pastoralist Community of Northeast Ethiopia Ethiop J Health Sci. Vol. 28, No. 4 July 2018	Afar	*To determine the prevalence of utilization of institutional delivery and associated factors.*	CS	women who gave birth in the last two years	1842	18.4	Low risk
21	Gebeyehu Dejene and Tesfahun Hailemariam, Utilization of Institutional Delivery Services and Associated Factors among Mothers in Semi-pastoralist, Southern Ethiopia J Women’s Health Care 2015, 4:7	Southern Nation Nationality and Peoples Region	To determine the prevalence of Institutional Delivery Services utilization and Associated Factors among Mothers in Semi-pastoralist, Southern Ethiopia	CS	women who gave birth in the last two years	756	14.6	Low risk
22	Asmamaw Limenih, Negussie Deyesa and Adugnaw Berhane,Assessing the Magnitude of Institutional Delivery Service Utilization and Associated Factors among Mothers in Debre Berhan, Ethiopia J Preg Child Health 2016, 3:3	Amhara	ssessing the magnitude of institutional delivery service utilization and associated factors.	CS	women who gave birth in the last two years	404	80.1	Low risk
23	Taye Shigute, Solomon Tejineh and Legesse Tadesse, Institutional Delivery Service Utilization and Associated Factors among Women of Child Bearing Age at Boset Woreda, Oromia Regional State, Central Ethiopia J Women’s Health Care 2017, 6:5	Oromiya	*To assess institutional delivery magnitude and associated factors among mothers delivered in the past 12 months in Boset Woreda, Ethiopia.*	CS	women who gave birth in the last one years	589	60.3	Low risk
24	Serawit Mekonnen Jinka, Legesse Tadesse Wodajo and Gebi Agero, Predictors of institutional delivery service utilization,among women of reproductive age group in DimaDistrict, Agnua zone, Gambella, Ethiopia. Medical Practice and Review Vol. 9(2), pp. 8–18	Gambiela	*Assessing institutional delivery and predictors in Gambella, Ethiopia*	CS	women who gave birth in the last one years	798	63.2	Low risk

**Key:-**CS=Cross-sectional; CC=Case Control

### Quality of studies

Based on Joanna Briggs Institute (JBI) quality appraisal checklist all the studies included in this systematic review and meta-analysis had low risk. Therefore, all of the studies were considered [24–47] **(**Table 1**).**

### Meta-analysis

#### Heterogeneity of studies

Heterogeneity test for the proportion of the review indicated *I*^2^ = 0.0%, and no variation was observed among the included studies hence fixed effect model was assumed in the analysis.

#### Prevalence of institutional delivery service utilization

The prevalence of institutional delivery service utilization of individual studies ranged from 12.1 to 80.1% [29, 30].The seven included studies from Amhara region, which were conducted at different periods of time showed that the prevalence estimates of institutional delivery service utilization were 74.7, 34.0, 62.3, 78.8, 15.7, 12.1, and 80.1% [24–30].The nine included studies from Oromiya region, which were conducted at different periods of time, showed that the prevalence of institutional delivery service utilization were 28.7,13.9,34.2,39.7,12.3,47.0,18.2,16.4, and 60.3% [31–39]. Three included studies from SNNPR region, which were conducted at different period of time, showed that the prevalence of institutional delivery service utilization was 14.6, 31.0 and 78.3% [43–45]. Two included studies from the Afar region, which was conducted at different period of time, showed that the prevalence of institutional delivery service utilization was 35.2 and 18.4% [40, 41]. Three different studies from Benishangul-Gumuz, Gambella, and Tigray and Oromiya showed that the prevalence of institutional delivery service utilization to be 51.1, 63.2 and 25.0% respectively [42, 46, 47].

**Fig. 4:**
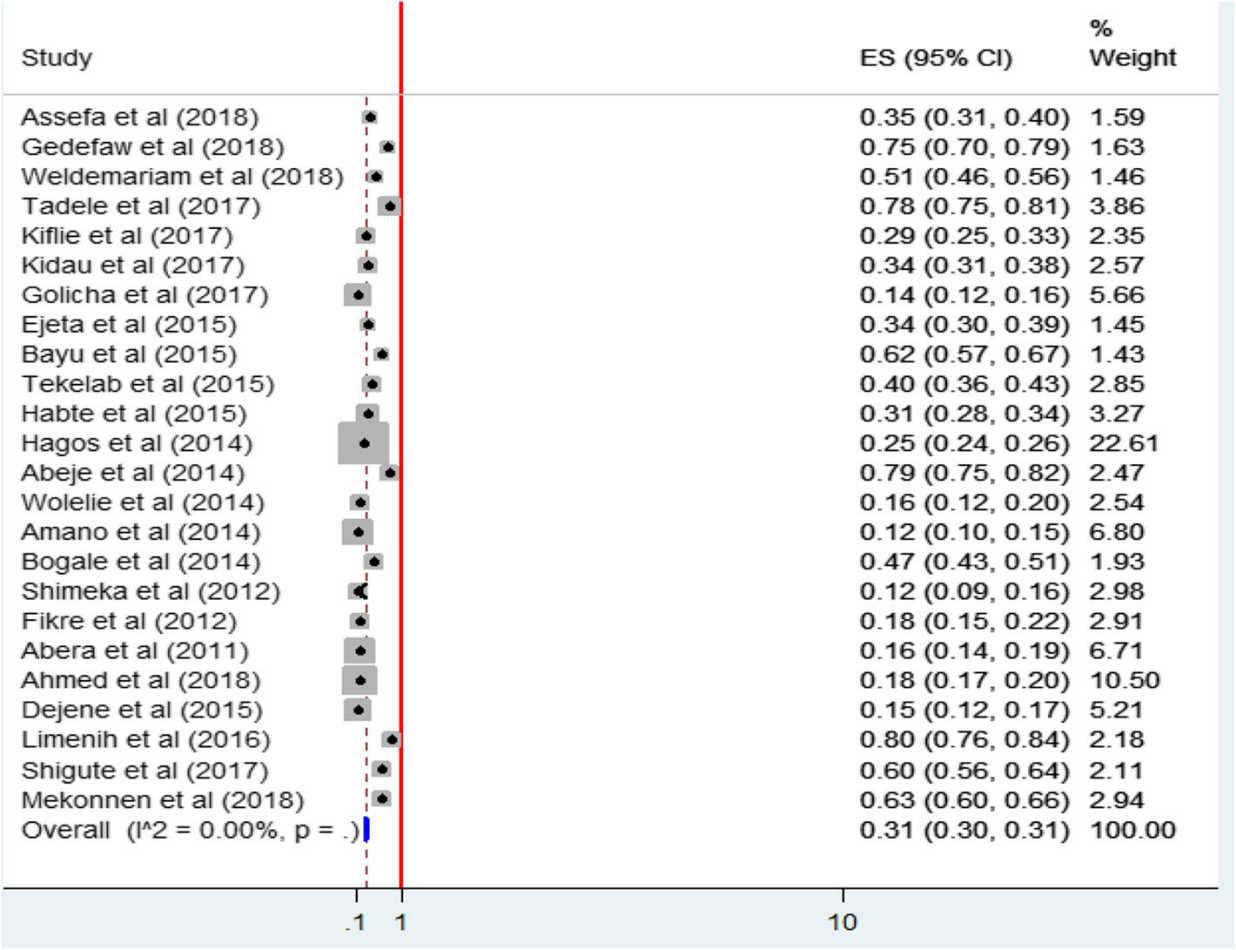
Forest plot of the Proportion of institutional delivery service utilization with corresponding 95% CIs

The estimated overall prevalence of institutional delivery service utilization is presented in a forest plot **(**Fig. 4**).** The overall pooled prevalence of institutional delivery service utilization was 31% (95% CI, 30, 31.2%, I^2^ = 0.000%).

#### Publication bias

**Fig. 5:**
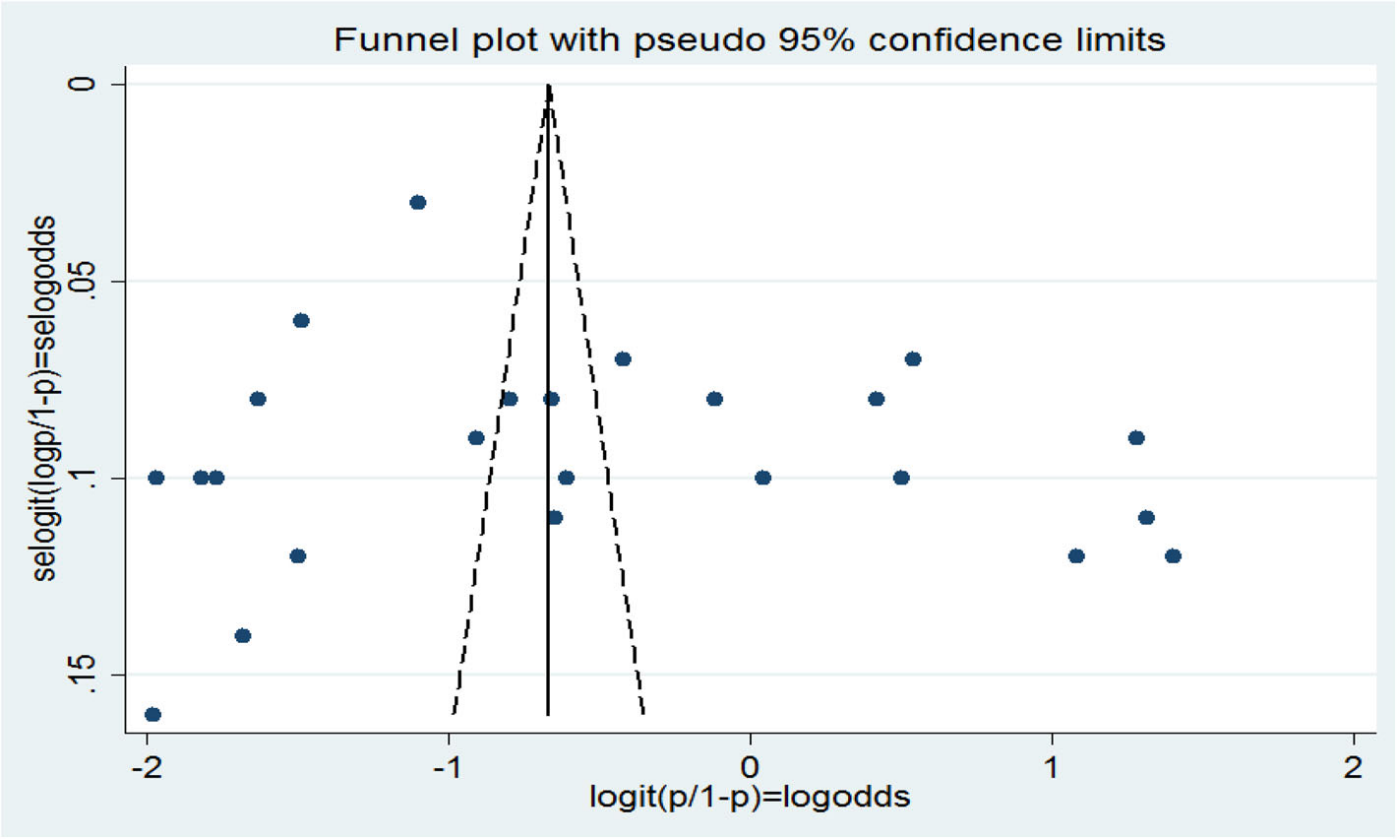
Funnel plot for publication bias

The publication bias could be assessed by using either a funnel plot (subjectively) or Eggers regression test (objectively).For this review, a funnel plot showed a symmetrical distribution **(**Fig. 5**).** Egger’s regression test *p*-value was 0.193, which indicated the absence of publication bias.

#### Subgroup analysis

The subgroup analysis was done based on the region and year of publication. Based on this, the prevalences of institutional delivery service utilization were found to be 47, 39 and 24% in Amhara, SNNPR and Oromiya studies, respectively. On the other hand the prevalences of institutional delivery service utilization were 37% between the year of 2015–2016 and 2017–2018, and 24% between the years of 2011–2014 **(**Table 2**).**

**Table 2:** The pooled proportion of IDSU, 95% CI and heterogeneity estimate with a p-value for the subgroup analysis

Variables	Characteristics	No of studies	Pooled proportion	(95% CI)	Weight	I^2^ (P-value)
By region (Fixed)	Afar	2	0.21	0.19 0.22	12.09	0.00%(0.000)
	Amhara	7	0.47	0.46 0.49	15.81	0.00%(0.000)
	Bie.Guz	1	0.51	0.46 0.56	1.46	0.00%(0.000)
	Southern Nation Nationality and Peoples Region	3	0.39	0.37 0.40	12.33	0.00%(0.000)
	Oromiya	9	0.24	0.23 0.25	32.77	0.00%(0.000)
	Tigray&Oromiya	1	0.25	0.24 0.26	22.61	0.00%(0.000)
	Gambiela	1	0.63	0.60 0.66	2.94	0.00%(0.000)
	Over all	24	0.31	0.30 0.31	100.00	0.00%(0.000)
By year of Publication (Fixed)	2017–2018	10	0.37	0.36 0.38	34.66	0.00%(0.000)
	2015–2016	6	0.37	0.35 0.38	16.39	0.00%(0.000)
	2011–2014	8	0.24	0.23 0.25	48.95	0.00%(0.000)
	Over all	24	0.31	0.30 0.31	100.00	0.00%(0.000)

#### Sensitivity analysis

The sensitivity analysis indicated that there was no study out of the confidence bound; all the studies had almost equal influence on the pooled proportion **(**Fig. 3**).**

#### Time-trend analysis

**Fig. 6:**
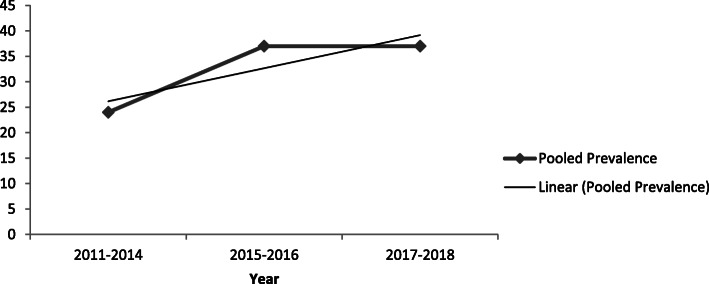
Time-trend analysis of the pooled prevalence of Institutional delivery service utilization in Ethiopia from 2011 to 2018

The time-trend analysis showed that the prevalence of institutional delivery service utilization has increased from 24% (95%CI 23, 25%) in 2011–2014 to 37%(95%CI 36, 38%) in 2017–2018. However, the pooled prevalence from year to year is increasing significantly (*p*-value ≤0.001) (Fig. 6).

#### Associated factors

This review indicated that institutional delivery service utilization in Ethiopian was associated with the three dynamic factors of Andersen Healthcare Utilization conceptual model, i.e. predisposing factors (the socio-cultural characteristics of individuals that exist prior to their illness.), enabling factors (the logistical aspects of obtaining care), and need factors (the most immediate cause of health service use, from functional and healthy problems that generate the need for health care services).

#### Predisposing factors

Women from a family where husbands had been a decision makers on a place where to give birth is 54% (AOR = 46%; 95% CI 18, 119%) less likely to give birth at health institution [33].

#### Attitude towards institutional delivery

Women who had a favorable attitude towards institutional delivery service utilization were 2.83 times (AOR = 2.80; 95% CI 1.60, 4.91) as likely to use institutional delivery service as compared to those to those women who had no favorable attitude [37].

**Fig. 7:**
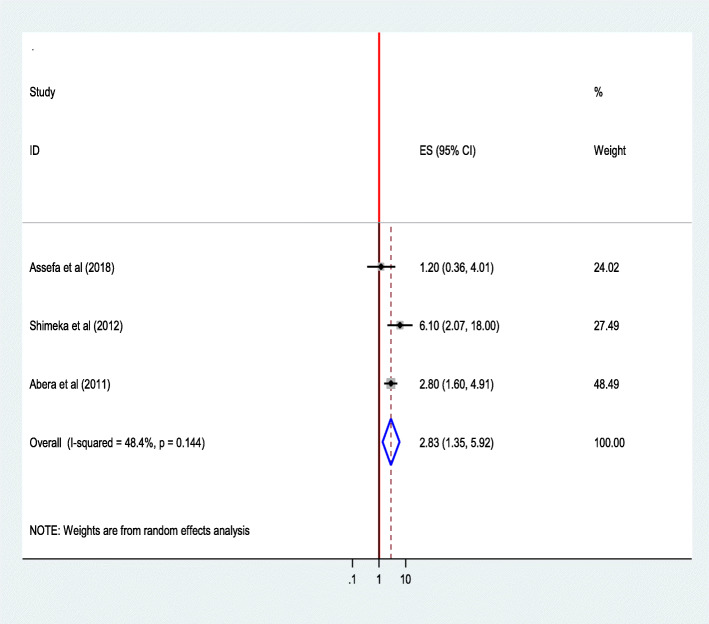
The pooled effects of maternal attitude on Institutional delivery service utilization

The pooled effect of three studies [29, 37, 40] showed that favorable attitude towards institutional delivery was a significant predictor of institutional delivery service utilization, and those mothers who had a favorable attitude towards institutional delivery service utilization were 2.83 times as likely to use the services. The heterogeneity test indicated moderate variability, *I*^2^ = 48.4% i.e. moderate heterogeneity, hence the random effect model was assumed in the analysis [48]. Sensitivity analysis indicated that no change was observed in the overall ORs **(**Fig. 7**).**

#### Maternal knowledge

The probability of utilizing health institution for delivery service was higher for those who had better knowledge on the danger signs of pregnancy and the benefits of institutional delivery service. Women who had good knowledge on institutional delivery service utilization were 2 times (AOR = 2.10; 95% CI 1.08, 4.09) as likely to use institutional delivery service as compared to those women with poor knowledge [40].

**Fig. 8:**
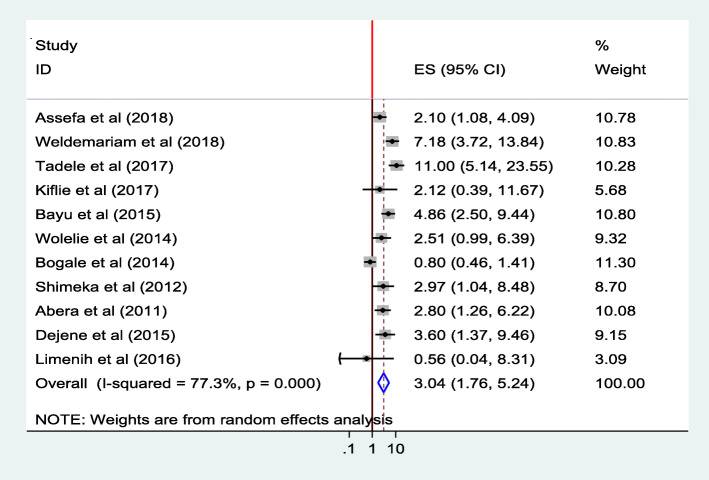
Association of knowledge of women’s with institutional delivery service utilization in Ethiopia, 2010–2018. Abbreviations: CI, confidence interval; *df*, degrees of freedom; D–L, Dersimonian and laird

The pooled effect of eleven studies [26, 28–31, 36, 37, 40, 42, 43, 45] showed that women who were knowledgeable were 3 times (AOR = 3.04;95%CI 1.76,5.24) as likely to give birth in a health institution than those women who were not knowledgeable. The heterogeneity test indicated high variability, *I*^2^ = 77.3%, i.e. high heterogeneity, hence random effect model was assumed in the analysis. Sensitivity analysis indicated that no change was observed in the overall ORs (Fig. 8).

#### Maternal age at first pregnancy

Women whose age was from 15 to 24 (AOR = 4.02; 95%CI 2.07–8.55) and 25–34 (AOR = 2.21; 95% CI 1.32–3.69) at first pregnancy were more likely to use institutional delivery service [34].

**Fig. 9:**
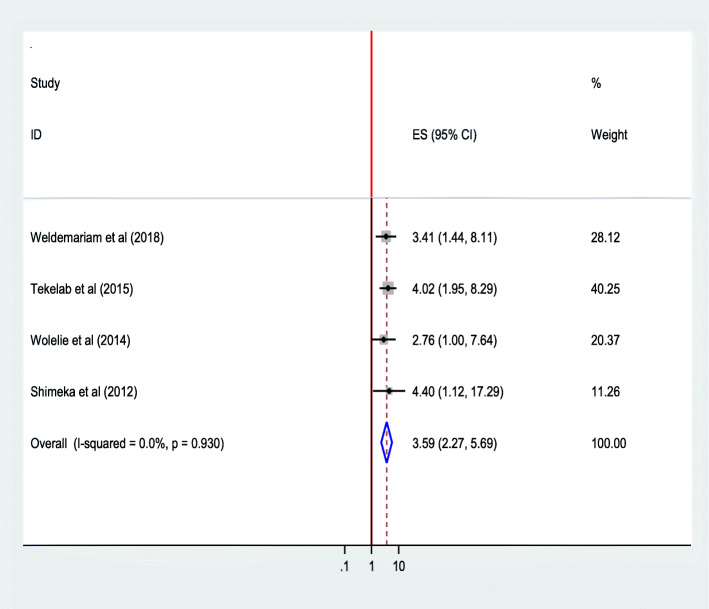
Association of age at first pregnancy (15-24 yrs) with institutional delivery service utilization in Ethiopia, 2010–2014.Abbreviations: CI, confidence interval; *df*, degrees of freedom Inverse Variance

**Fig. 10:**
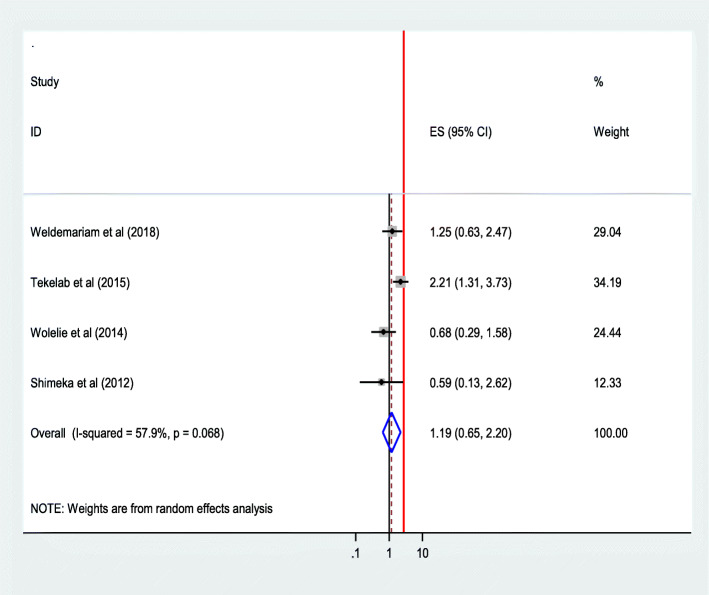
Association of age at first pregnancy (25-34 yrs) with institutional delivery service utilization in Ethiopia, 2010–2018. Abbreviations: CI, confidence interval; *df*, degrees of freedom; Inverse Variance

The pooled effect of four studies [28, 29, 34, 42] showed that women who had their first pregnancy between the age of 15–24 years were 3.60 times as likely to give birth in a health institution than those who became pregnant after 35 years of age (AOR = 3.59; 95% CI 2.27 5.69). The heterogeneity test indicated *I*^2^ = 0.0%, no variability was observed among the included studies hence fixed effect model was assumed in the analysis. Sensitivity analysis illustrated stability of overall ORs [48] (Fig. 9).Where as women who had their first pregnancy between the ages of 25–34 years were not became significantly associated factors (Fig. 10).

#### Maternal education

This review indicated that there was significant association between women’s educational status and utilization of institutional delivery service. Women who can read & write (AOR = 1.75; 95% CI 1.21–2.54), those with primary level education (AOR = 2.23;95%CI 1.39,3.59) and those with secondary & above educational level (AOR = 2.40;95%CI 1.05,5.49) were more likely to use institutional delivery service [25].

**Fig. 11:**
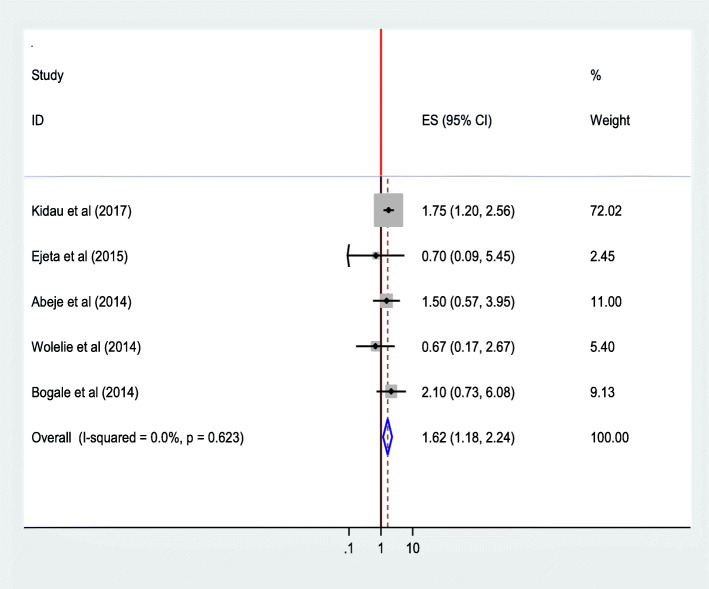
Association of educational statuses (can read and write) of the women with institutional delivery service utilization in Ethiopia, 2010–2018. Abbreviations: CI, confidence interval; *df*, degrees of freedom;I-V,Inverse Variance

The pooled estimate of five studies [25, 27, 28, 33, 36] also indicated a significant association between mothers’ educational status and utilization of institutional delivery service. Mothers who can read and write were 1.62 times as likely to give birth at the health institution as compared to those who can’t read& write(AOR = 1.62;95%CI 1.18–2.24). Heterogeneity test indicated *I*^2^ = 0.0%, and hence fixed effect model was assumed in the analysis. Sensitivity analysis did not bring significant change in the overall ORs (Fig. 11).

**Fig. 12:**
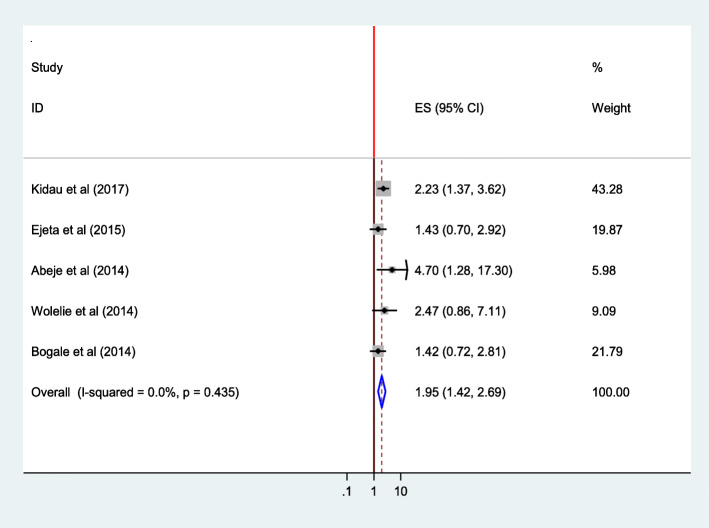
Association of educational statuses (Primary school) of the women with institutional delivery service utilization in Ethiopia, 2010–2018. Abbreviations: CI, confidence interval; *df*, degrees of freedom; Inverse Variance

Similarly, women’s who attend primary school were almost two times as likely to give birth at the health institution than who can’t read and write (AOR = 1.95; 95% CI 1.42–2.69). Heterogeneity test indicated *I*^2^ = 0.0%, and hence fixed effect model was assumed in the analysis. Sensitivity analysis did not bring significant change in the overall ORs (Fig. 12).

**Fig. 13:**
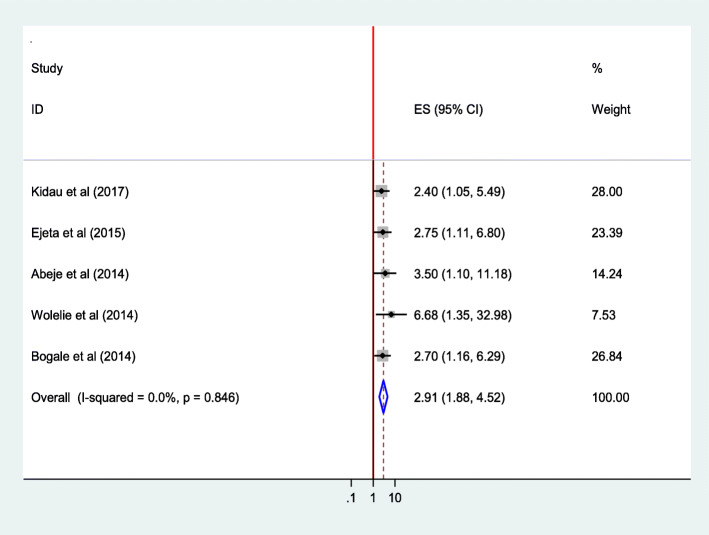
Association of educational statuses (Secondary school & above) of the women with institutional delivery service utilization in Ethiopia, 2010–2018. Abbreviations: CI, confidence interval; *df*, degrees of freedom; I-V,inverse variance

Women who attended secondary and above educational level were almost three times as likely to give birth at health institution as compared to women who can’t read and write (AOR = 2.92;95% 1.88–4.52. Heterogeneity test indicated *I*^2^ = 0.0%, and hence fixed effect model was assumed in the analysis. Sensitivity analysis did not bring significant change in the overall ORs (Fig. 13).

#### Maternal occupation

Women who were not house wives (AOR = 2.50; 95% CI 0.75,8.30) were more likely to use institutional delivery service [27].

**Fig. 14:**
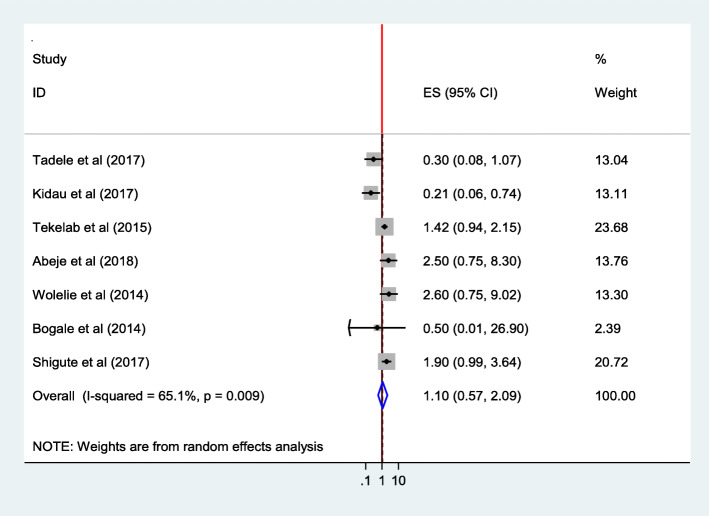
Association of Occupation of the women with institutional delivery service utilization in Ethiopia, 2010–2018. Abbreviations: CI, confidence interval; *df*, degrees of freedom; D–L, Dersimonian and laird

The pooled estimate of five studies [25, 27, 28, 34, 36, 38, 43] showed insignificant association between mothers’ occupation and utilization of institutional delivery service (AOR = 1.10; 95% CI 0.57,2.09). Heterogeneity test indicated *I*^2^ = 65.1%, moderate variability, and hence the random effect model was assumed during analysis. Sensitivity analysis was done, and no change was noted on overall OR (Fig. 14).

#### Parity

**Fig. 15:**
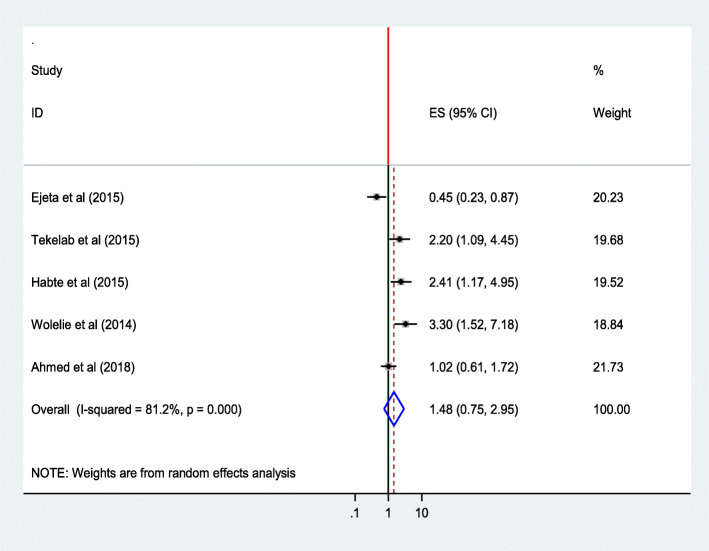
Association of parity (Parity = 1) with institutional delivery service utilization in Ethiopia, 2010–2018. Abbreviations: CI, confidence interval; *df*, degrees of freedom;D-L, D–L, Dersimonian and laird

The combined estimate of five studies [28, 33, 34, 41, 44] showed that the number of children women delivered was not significantly associated with institutional delivery service utilization (AOR = 1.48; 95% CI 0.75,2.95) and (AOR = 1.10;95%CI 0.87,1.39) for women who have one child and 2-4children respectively. The heterogeneity test indicated for women who have one child is *I*^2^ = 81.2%, and hence the random effect model was assumed in analysis. Sensitivity analysis was done, and no significant change was observed in overall OR (Fig. 15), whereas for women who have 2–4 children I^2^ = 0.0%, and hence fixed effect model was assumed in the analysis.

### Enabling factors

#### Availability of information source

**Fig. 16:**
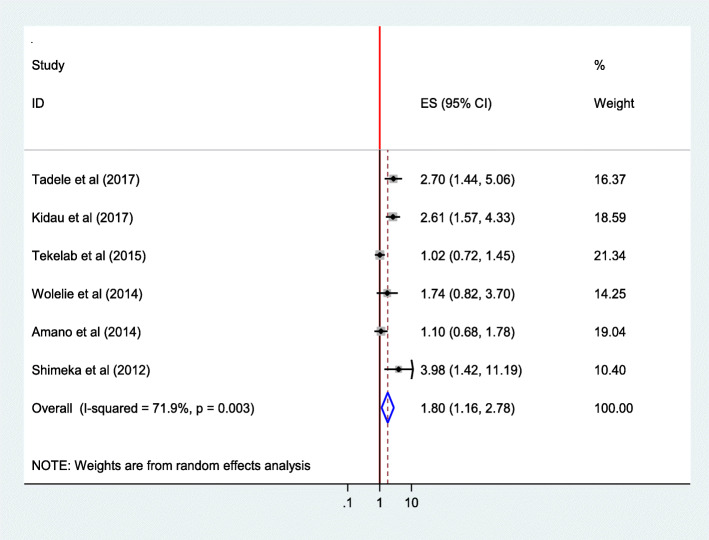
Association of availability of information source with institutional delivery service utilization in Ethiopia, 2010–2018. Abbreviations: CI, confidence interval; *df*, degrees of freedom;D-L, D–L, Dersimonian and laird

The chance of delivering in health institution among those women with access to information source was1.8 times higher than those women with no access to information source (AOR = 1.80; 95%CI 1.16, 2.78).The heterogeneity test indicated *I*^2^ = 71.9%, and hence the random effect model was assumed in the analysis. Sensitivity analysis revealed the stability of overall effect size (Fig. 16).

#### Place of residence

**Fig. 17:**
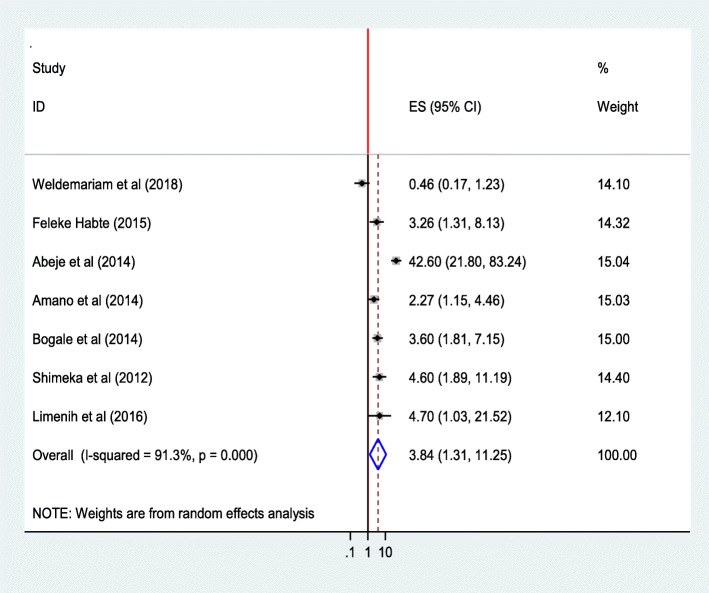
Association of place of residence with institutional delivery service utilization in Ethiopia, 2010–2018. Abbreviations: CI, confidence interval; *df*, degrees of freedom;D-L, D–L, Dersimonian and laird

The pooled estimate of seven studies [27, 29, 30, 35, 36, 42, 44] showed that place of residence defined as rural and urban were the enabling factors that determined utilization of institutional delivery service. Women from urban area were 3.84 times as likely to deliver at a health institution than women from rural areas (AOR = 3.84; 95%CI 1.31, 11.25).The heterogeneity test indicated *I*^2^ = 91.3%, hence the random effect model was assumed in the analysis. Sensitivity analysis illustrated no change in the overall OR **(**Fig. 17**).**

#### Distance to health facility

**Fig. 18:**
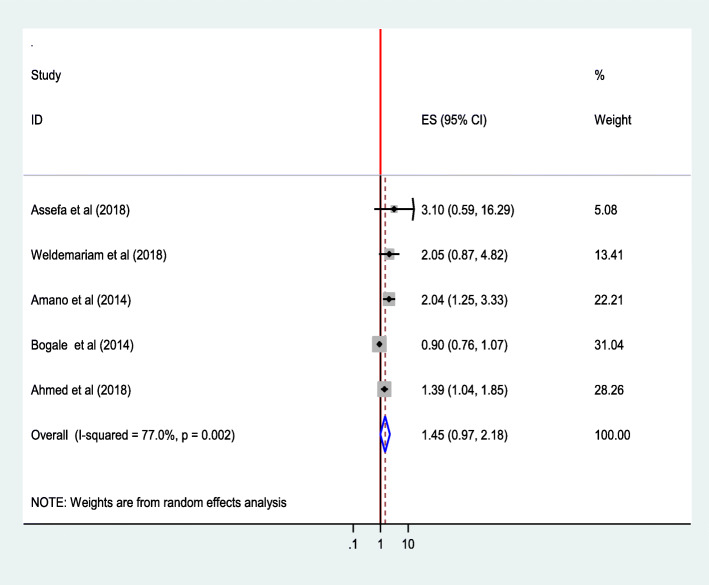
Association of distance to health facility with institutional delivery service utilization in Ethiopia, 2010–2018. Abbreviations: CI, confidence interval; *df*, degrees of freedom;D-L, D–L, Dersimonian and laird

The findings of five studies [28, 33, 34, 41, 44] showed that distance from a health facility was not significantly associated with institutional delivery service utilization (AOR = 1.45;95% CI 0.97,2.18).The heterogeneity test indicated *I*^2^ = 77%, and hence the random effect model was assumed in the analysis. Sensitivity analysis showed no significant change in overall ORs (Fig. 18).

### Need factors

#### ANC follow up

**Fig. 19:**
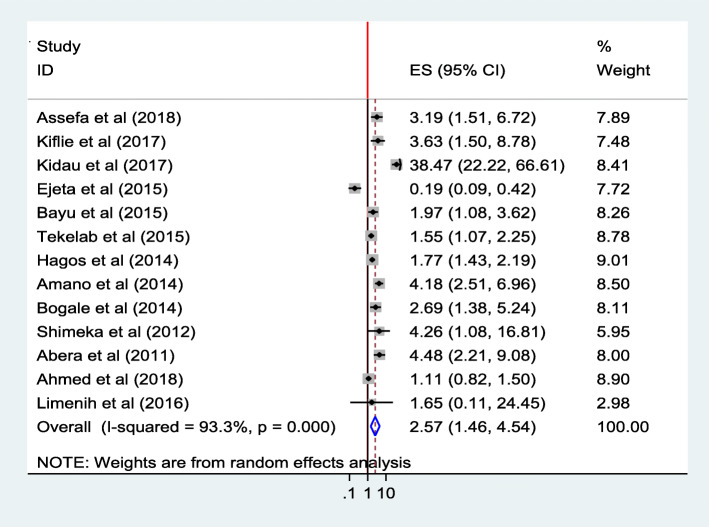
Association of ANC follow up with institutional delivery service utilization in Ethiopia, 2010–2018. Abbreviations: CI, confidence interval; *df*, degrees of freedom;D-L, D–L, Dersimonian and laird

The combined finding of thirteen studies [25, 26, 29–31, 33–37, 40, 41, 47] showed that ANC follow up was significantly associated with institutional delivery service utilization. Women who had ANC follow-up were 2.6 times (AOR = 2.57; 95% CI 1.46, 4.54) as likely to utilize the service than those who did not have ANC follow up. The heterogeneity test indicated *I*^2^ = 93.3%, and hence the random effect model was assumed in the analysis. The heterogeneity test indicated *I*^2^ = 93.3%, hence the random effect model was assumed in the analysis. Sensitivity test indicated no change in the overall ORs (Fig. 19).

#### Frequency of ANC follows up

**Fig. 20:**
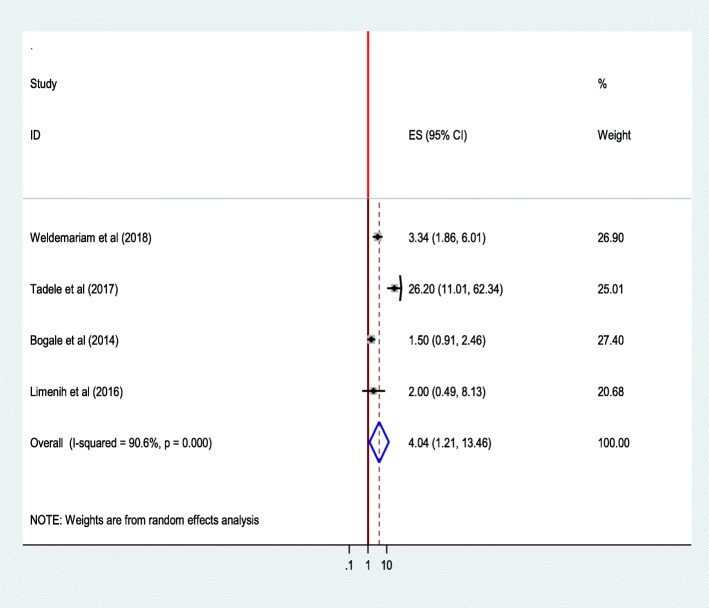
Association of Frequency of ANC follow up with institutional delivery service utilization in Ethiopia, 2010–2018. Abbreviations: CI, confidence interval; *df*, degrees of freedom;D-L, D–L, Dersimonian and laird

The combined effect of four studies [30, 36, 42, 43] showed that attending four or more ANC follow ups was significantly associated with institutional delivery service utilization. Women who attended ANC for four or more times were 4 times as likely to give birth in a health institution than those who attended less than four times (AOR = 4.04;95%CI 1.21,13.46). Heterogeneity test indicated *I*^2^ = 90.6%, and hence random effect model was assumed in the analysis. Sensitivity test demonstrated stability of the overall ORs (Fig. 20).

#### Place of birth of the most recent birth

**Fig. 21:**
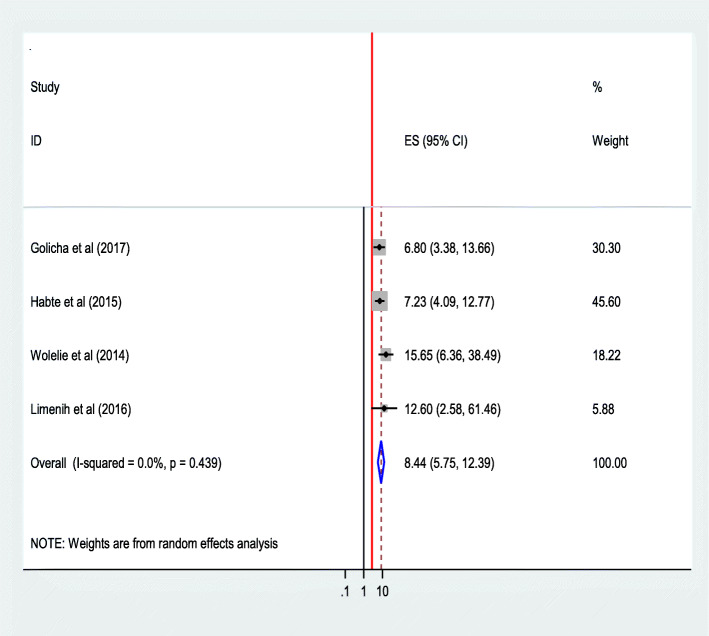
Association of Place of birth the most recent birth of the women with institutional delivery service utilization in Ethiopia, 2010–2018. Abbreviations: CI, confidence interval; *df*, degrees of freedom;I-V,inverse variance

The pooled effect of four studies [28, 30, 32, 44] showed that women who delivered their most recent birth at a health institution were 8.44 times as likely to utilize the service again than those who gave the most recent birth in home (AOR = 8.44;95% CI 5.75,12.39).The heterogeneity test indicated *I*^2^ = 0.0%, no heterogeneity, and hence the fixed effect model was assumed in the analysis. Sensitivity analysis did not bring significant change in the overall OR **(**Fig. 21**).**

#### Presence of complication during birth preceding the most recent birth

**Fig. 22:**
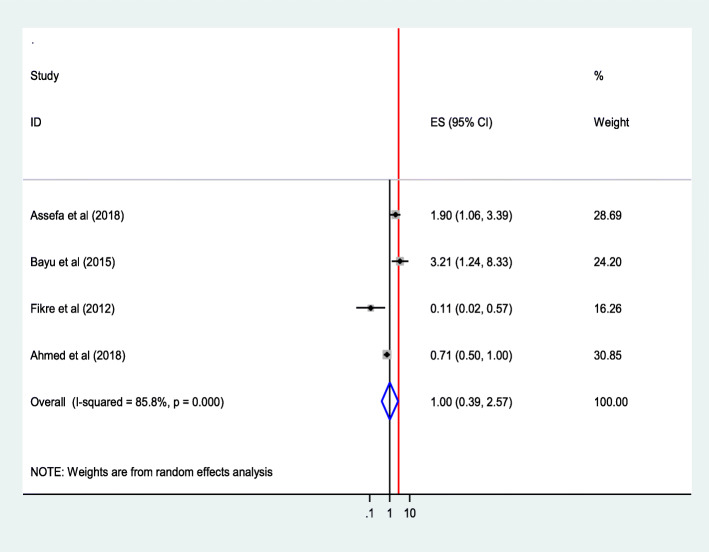
Association of Presence of complication during birth preceding the most recent birth with institutional delivery service utilization in Ethiopia, 2010–2018. Abbreviations: CI, confidence interval; *df*, degrees of freedom;D-L, D–L, Dersimonian and laird

The finding of review of four studies [26, 39–41] indicated that the presence of complication during birth preceding the most recent birth was not significantly associated with institutional delivery service utilization (AOR = 1.00; 95%CI 0.39, 2.70).The heterogeneity test indicated *I*^2^ = 85.8%, high variability, and hence the random effect model was assumed in the analysis. Sensitivity analysis illustrated stability of overall OR (Fig. 22).

## Discussion

In this review, twenty four studies comprising a total of 19,969 participants were analyzed to estimate the best available evidence for the prevalence and factors associated with institutional delivery service utilization in Ethiopia. The findings of the review revealed valuable information which is comparable with all the factors related to the outcome variable across the nation. The factors were related to the three dynamic factors of Andersen Healthcare Utilization conceptual model, i.e. predisposing factors (the socio-cultural characteristics of people that exist before their illness), enabling factors (the logistical aspects of obtaining care), and wish factors (the most immediate explanation for health service use, from functional and health problems that generate the necessity for health care services).

This meta-analysis estimated the national prevalence of institutional delivery service utilization in Ethiopia. Accordingly, the national pooled prevalence of institutional delivery service utilization was 31% (95% CI: 30–31.2%). This result was in line with an analysis study of Four Demographic surveillance sites of South Asia [49].But less than a study from Haiti 2012 Demographic Health Survey and 2013 service provision assessment survey (39%) [50] and an analysis of Nepal Demographic and Health survey 2011(36.9%) [51].This difference is probably due to the differences in awareness creation regarding institutional delivery service for the large community, and community engagement and access to health services.

In this review, the prevalence of institutional delivery service utilization was high in Amhara as compared to other regions. Those studies included in this meta-analysis were conducted in the community based settings.

Based on the pooled analysis of three or more AOR of studies, maternal age at first pregnancy (15–24 years), place of residence (Urban), maternal knowledge (good knowledge), maternal educational status (Can read and write, primary level, secondary and above),attitude towards institutional delivery (favorable attitude), availability of information system, ANC follow- up, the frequency of ANC (4+) follow-up and place of delivery the most recent birth (health institution) were associated with institutional delivery service utilization.

The odds of giving birth at a health facility were higher among women whose age was from 15 to 24 years. This finding is in line with primary studies conducted in Nepal and three districts of Tanzania [51, 52].Younger women are more likely to be literate and have a better knowledge on the benefits of institutional delivery than older women. This could have helped them choose to give birth at a health facility. In addition to that home delivery may not be considered as risky for most of the older women who have previously experienced birth at home.

Another important predictors of institutional delivery service utilization from this review was women’s knowledge on danger sign of pregnancy and the benefit of institutional delivery service utilization (AOR = 3.04; 95%CI 1.76, 5.24). This finding was in agreement with primary study findings from Kenya [53] and Southern Tanzania that showed the increase of skilled birth attendance when women have knowledge about risks during pregnancy (AOR = 2.95; 95% CI 1.65,5.25) [54]. Knowledge is a crucial factor that affects attitude, intention and behavior. Women who have sufficient knowledge about danger signs of pregnancy might have perceived service benefits of a health institution, like complication management by skilled health care workers in time of labor.

Educational status of women was found to be one of the predictors in this review. Women who can read & write, attended primary, secondary & above education were likely to use institutional delivery service as compared to women who can’t read & write. This finding is consistent with studies from other countries including: an analysis of the Four Demographic surveillance sites of South Asia [49], from 2012 Haiti demographic Health Survey & 2013 Haiti service provision assessment survey [50], Guatemala, Mexco and panama [55], an analysis from Bangladesh Demographic Health Survey 2011 [56], and an analysis of 2013 Nigeria DHS data set [57] and a primary study in Guinea [58]. Education makes mothers to be more concerned for institutional delivery service utilization, keeping their health status in regular way, and will have sense of ownership, ability and freedom to make decisions about their own health is more favorable. On the other hand, education improves the ability of women to afford the cost of medical health care service, which ultimately enhances their health-seeking behavior.

The exposure status with media have a significant role on institutional delivery service utilization in which women with adequate exposure to the media had more odds of institutional delivery than women with inadequate exposure. This finding was supported by a primary cross sectional study in a remote mountain district of Nepal [59]. The utilization of institutional delivery service might be high when women have adequate exposure to the media since media exposure would increase the women’s concern and awareness of pregnancy related issues, increase their familiarity with medical personnel which in turn expose the women to more health education, counseling and the need for professional help.

Place of the women’s residence was significantly associated with the utilization of institutional delivery service. This finding was consistent with an analysis of the 2011 Demographic and Health Survey of Nepal, which showed that urban and rural differences had significant associations with institutional delivery service utilization (AOR = 2.42; 95%CI 1.83 3.19) [51].It is also in agreement with the analysis of 2011 Bangladeshi Demographic and Health Survey (AOR = 1.84) [56], and a systematic review conducted in Ethiopia (AOR = 13.16) [48]. The possible explanation might be differences in the characteristics of the urban residents i.e. there might be more educated mothers, might have better knowledge of institutional delivery service, more accessibility and availability of health care services, and better access to information via different media than rural mothers.

The result of this review revealed that attendance of four or more antenatal visits was significantly associated with health facility delivery. This finding is consistent with primary studies conducted in Kenya [60], Tanzania [61], an analysis of 2013 Nigeria DHS data set [57], an analysis of Nepal Demographic and Health survey 2011 [51], 2011 Bangladeshi Demographic Health survey (AOR = 3.639) [56] and a systematic review conducted in Ethiopia(AOR = 3.24) [48]. Having four or more ANC visits is the recommended ANC visit by WHO and which might reflect the woman’s concern of her pregnancy related issues and the need for professional help and visiting ANC frequently increasing their familiarity with medical personnel which expose the women to more health education and counseling which are more likely to increase the utilization of delivery service [48].

### Policy implications

This review suggests that increasing women’s participation in different health and health care activities will have a long-term positive effect on institutional delivery service utilization. This review also identifies the need to do with the attitude towards institutional delivery, residence, and age specific intervention activity; and provide education and counseling to pregnant woman and their partner about the benefits of delivering in a health facility, the potential risks and complications associated with pregnancy in general, and home delivery in particular. Promotion of institutional delivery service and prevention of home delivery should be an agenda in the healthcare system of the country. We also recommend further research to be conducted on the community level of readiness on the promotion of institutional delivery service utilization and prevention of home delivery, and women’s perceptions towards institutional delivery services.

### Strength and limitation

Focusing on women who had given birth within the 2 years preceding the survey to be included in this review is one of the strengths of this review since women might not have been subject to recall bias within this period. We also conducted a methodologically rigorous meta-analysis, reported an adjusted odds ratio, sensitivity analysis and sub-group analysis, and used quality indicators to select only sound publications to ensure the quality of the research findings. However, in this review, 90% of the included studies were cross-sectional in nature which limited our ability to assess the cause-effect relationship. We were not also able to show the pooled estimates for all variables associated with institutional delivery because the included studies classified the variables in different ways.

## Conclusion

This systematic review and Meta-analysis revealed institutional delivery service utilization remains low in Ethiopia. It also showed that attitude towards institutional delivery, maternal age at first pregnancy, residence setting, educational status of mothers, availability of information source, ANC follow-up, frequency of ANC follow up, knowledge on the benefits of institutional delivery & danger signs of pregnancy and place of delivery for the most recent birth were factors positively and significantly associated with institutional delivery service utilization. However, maternal occupation, parity, distance from a health facility and presence of complication during birth preceding the most recent birth were not associated with institutional delivery service utilization. This review may help policy-makers and program officers to design appropriate interventions to increase institutional delivery service utilization.

## Supplementary information

**Additional file 1.** Quality appraisal of included study.

## Data Availability

The datasets generated and/or analyzed during the current review study are available at University of Gondar, College of medicine and Health Science, Institute of Public Health repository [www.UoG.edu.et] in soft copy. In addition the data are available from the authors upon reasonable request and with permission of the principal investigators (Adane Nigusie- E-mail adane_n@yahoo.com, Adane.Nigusie@uog.edu.et).

## Abbreviations

ANC: Antenatal car
AOR: Adjusted Odd Ratio
CI: Confidence interval
DF: Degree of freedom
D-L: Dersimonian and laird
EDHS: Ethiopia Demographic and Health Survey
IDUS: Institutional delivery service utilization
I-V: Inverse variance
JBI: Joanna Briggs Institute
OR: Odds Ratio
PRISMA: Preferred Reporting Items for Systematic Review and Meta-Analysis statement
SDG: Sustainable Development Goals
SNNPR: Southern Nation Nationality and Peoples Region
