# Supplementary material for: Institutional delivery service utilization and associated factors in Ethiopia: a systematic review and META-analysis
Source: BMC Pregnancy Childbirth. 2020 Jun 15;20:364. doi: 10.1186/s12884-020-03032-5 (PMC7296650; doi:10.1186/s12884-020-03032-5)
Supplement: Supplementary file 1 — Additional file 1. Quality appraisal of included study. [file 12884_2020_3032_MOESM1_ESM.docx]

**Quality appraisal of included study**

| Included studies | Eight JBI Critical Appraisal Checklist for Cross Sectional Studies(Yes,No,Unclear)  If Yes(>=50%).....low risk(two raters).....>1=Yes 2=No 3= Unclear | | | | | | | | | | | | | | | | |
| --- | --- | --- | --- | --- | --- | --- | --- | --- | --- | --- | --- | --- | --- | --- | --- | --- | --- |
|  | Inclusion criteria | | Description of study subject and setting | | Valid and reliable measurement of exposure | | Objective and standard criteria used | | Identification of confounder | | Strategies to handle confounder | | Outcome measureme  nt | | Appropriate statistical analysis | | Over all appraisal |
|  | R1 | R2 | R1 | R2 | R1 | R2 | R1 | R2 | R1 | R2 | R1 | R2 | R1 | R2 | R1 | R2 |  |
| 1.Assefa et al | 1 | 1 | 1 | 1 | 1 | 1 | 1 | 1 | 1 | 2 | 1 | 1 | 1 | 1 | 2 | 1 | 0.875 |
| 2.Gedefaw et al | 1 | 2 | 1 | 1 | 2 | 2 | 1 | 1 | 1 | 1 | 1 | 1 | 1 | 1 | 1 | 1 | 0.8125 |
| 3.Weldemariam et al | 1 | 1 | 1 | 1 | 1 | 1 | 1 | 1 | 1 | 1 | 1 | 1 | 1 | 1 | 1 | 1 | 1 |
| 4.Tadele et al | 2 | 1 | 1 | 1 | 2 | 2 | 1 | 1 | 1 | 1 | 1 | 1 | 1 | 1 | 2 | 2 | 0.6875 |
| 5.Kiflie et al | 1 | 1 | 1 | 1 | 1 | 1 | 1 | 1 | 1 | 1 | 1 | 1 | 1 | 1 | 2 | 2 | 0.875 |
| 6.Kidau et al | 1 | 1 | 1 | 1 | 1 | 2 | 1 | 1 | 2 | 2 | 2 | 1 | 1 | 1 | 1 | 1 | 0.75 |
| 7.Golicha et al | 2 | 2 | 1 | 1 | 1 | 2 | 2 | 2 | 1 | 1 | 1 | 1 | 1 | 1 | 1 | 1 | 0.6875 |
| 8.Tekelab et al | 1 | 1 | 1 | 1 | 1 | 1 | 1 | 1 | 2 | 2 | 2 | 1 | 1 | 1 | 1 | 1 | 0.8125 |
| 9.Habte et al | 2 | 2 | 1 | 1 | 1 | 2 | 2 | 2 | 1 | 1 | 1 | 1 | 1 | 1 | 1 | 1 | 0.6875 |
| 10.Hagos et al | 2 | 2 | 1 | 1 | 1 | 1 | 1 | 1 | 1 | 1 | 1 | 1 | 1 | 1 | 1 | 1 | 0.875 |
| 11.Abeje et al | 1 | 1 | 1 | 1 | 1 | 1 | 1 | 1 | 1 | 1 | 1 | 1 | 1 | 1 | 2 | 2 | 0.875 |
| 12.Wolelie et al | 2 | 1 | 1 | 1 | 1 | 1 | 1 | 1 | 1 | 1 | 1 | 1 | 1 | 1 | 1 | 1 | 0.9375 |
| 13.Amano et al | 1 | 1 | 1 | 1 | 1 | 1 | 1 | 1 | 2 | 2 | 2 | 1 | 1 | 1 | 1 | 1 | 0.8125 |
| 14.Bogale et al | 1 | 1 | 1 | 1 | 1 | 1 | 2 | 2 | 1 | 1 | 1 | 1 | 1 | 1 | 1 | 1 | 0.875 |
| 15.Shimeka et al | 1 | 1 | 1 | 1 | 1 | 1 | 1 | 1 | 2 | 2 | 2 | 1 | 1 | 1 | 1 | 1 | 0.8125 |
| 16.Fikre et al | 2 | 1 | 1 | 1 | 1 | 1 | 2 | 2 | 1 | 1 | 1 | 1 | 1 | 1 | 1 | 1 | 0.8125 |
| 17.Abera et al | 1 | 2 | 1 | 1 | 1 | 1 | 1 | 1 | 1 | 1 | 1 | 1 | 1 | 1 | 1 | 1 | 0.9375 |
| 18.Ahmed et al | 1 | 1 | 1 | 1 | 2 | 2 | 1 | 1 | 1 | 1 | 1 | 1 | 1 | 1 | 1 | 1 | 0.875 |
| 19.Dejene et al | 2 | 2 | 1 | 1 | 1 | 1 | 1 | 1 | 1 | 1 | 1 | 1 | 1 | 1 | 1 | 1 | 0.875 |
| 20.Limenih et al | 1 | 1 | 1 | 1 | 2 | 2 | 1 | 1 | 2 | 2 | 2 | 1 | 1 | 1 | 1 | 1 | 0.6875 |
| 21.Shigute et al | 1 | 1 | 1 | 1 | 1 | 1 | 2 | 2 | 1 | 1 | 1 | 1 | 1 | 1 | 1 | 1 | 0.875 |
| 22.Mekonnen et al | 2 | 1 | 1 | 1 | 1 | 1 | 1 | 1 | 1 | 2 | 1 | 1 | 1 | 1 | 1 | 1 | 0.875 |

| Included studies | **Ten** JBI Critical Appraisal Checklist for Case control Studies(Yes,No,Unclear)  If Yes(>=50%).....low risk(two raters).....>1=Yes 2=No 3= Unclear | | | | | | | | | | | | | | | | | | | | |
| --- | --- | --- | --- | --- | --- | --- | --- | --- | --- | --- | --- | --- | --- | --- | --- | --- | --- | --- | --- | --- | --- |
|  | 1 | | 2 | | 3 | | 4 | | 5 | | 6 | | 7 | | 8 | | 9 | | 10 | | Over all |
|  | R1 | R2 | R1 | R2 | R1 | R2 | R1 | R2 | R1 | R2 | R1 | R2 | R1 | R2 | R1 | R2 | R1 | R2 | R1 | R2 |  |
| Ejeta et al | 2 | 2 | 2 | 2 | 1 | 1 | 1 | 1 | 1 | 1 | 2 | 2 | 2 | 2 | 1 | 1 | 1 | 1 | 1 | 1 | 0.6 |

| Included studies | **Eleven** JBI Critical Appraisal Checklist for Cohort Studies(Yes,No,Unclear)  If Yes(>=50%).....low risk(two raters).....>1=Yes 2=No 3= Unclear | | | | | | | | | | | | | | | | | | | | | | |  |
| --- | --- | --- | --- | --- | --- | --- | --- | --- | --- | --- | --- | --- | --- | --- | --- | --- | --- | --- | --- | --- | --- | --- | --- | --- |
|  | 1 | | 2 | | 3 | | 4 | | 5 | | 6 | | 7 | | 8 | | 9 | | 10 | | 11 | | Over all |  |
|  | R1 | R2 | R1 | R2 | R1 | R2 | R1 | R2 | R1 | R2 | R1 | R2 | R1 | R2 | R1 | R2 | R1 | R2 | R1 | R2 | R1 | R2 |  | |
| Bayu et al | 1 | 1 | 1 | 1 | 1 | 1 | 1 | 1 | 2 | 2 | 1 | 1 | 1 | 1 | 1 | 1 | 1 | 1 | 1 | 1 | 1 | 1 | 0.818 | |
